# Reporting of pre-existing multiple long-term conditions in physical rehabilitation for long COVID: a scoping review

**DOI:** 10.1183/16000617.0123-2024

**Published:** 2024-11-27

**Authors:** Lucy Gardiner, Hannah M.L. Young, Holly Drover, Emily Morgan-Selvaratnam, Michael Natt, Nikki Smith, Enya Daynes, Mark W. Orme, Rod S. Taylor, Sally J. Singh, Rachael A. Evans

**Affiliations:** 1 Department of Respiratory Sciences, University of Leicester, Leicester, UK; 2 NIHR Leicester Biomedical Research Centre – Respiratory, University of Leicester, Leicester, UK; 3 University Hospitals of Leicester NHS Trust, Leicester, UK; 4 Diabetes Research Centre, University of Leicester, Leicester, UK; 5 Long COVID LTC Study Patient Advisory Group, Leicester, UK; 6 MRC/CSO Social and Public Health Sciences Unit and Robertson Centre for Biostatistics, University of Glasgow, Glasgow, UK

## Abstract

**Background:**

Physical rehabilitation may improve health and wellbeing outcomes for some adults living with long COVID. However, individuals living with pre-existing multiple long-term conditions (MLTCs) and long COVID may have additional rehabilitation challenges. This scoping review aims to identify the available evidence describing physical rehabilitation interventions for adults living with long COVID, to systematically map the reporting of pre-existing MLTCs, and to describe the characteristics of physical rehabilitation interventions used in adults with both pre-existing long-term conditions (LTCs) and long COVID.

**Methods:**

MEDLINE, CINAHL, Scopus, APA PsycInfo, medRxiv, OpenGrey and MedNar were searched from January 2020 to July 2023. Eligibility criteria included adults with long COVID, rehabilitation interventions including a physical component in any setting and any study design investigating interventions or intervention content except case series/reports.

**Results:**

Of 5326 unique records, 50 articles met the inclusion criteria, of which 25 (50%) made reference to pre-existing LTCs. These articles included four protocols and one consensus statement. Four of the remaining 20 studies (20%) reported the number of pre-existing LTCs, enabling the differentiation of individuals with MLTCs. One study reported outcomes of individuals with MLTCs separately to those without. The interventions described (k=24) typically consisted of combined aerobic and strength exercises (k=17 (71%)) in an outpatient setting (k=13 (54%)).

**Conclusions:**

There is limited and inconsistent reporting of the presence of MLTCs in studies of physical rehabilitation for adults with long COVID. Clarity and consistency of reporting of MLTCs is required to enable evaluation and adaptation of interventions to improve health and wellbeing for this population.

## Shareable abstract

**In physical rehabilitation studies for long COVID, reporting of pre-existing long-term conditions is limited and variable. Consistent and appropriately detailed reporting is required to enable evaluation of rehabilitation interventions in this population.** https://bit.ly/4grwpLf

## Introduction

Multiple long-term conditions (MLTCs) or “multimorbidity”, defined as two or more long-term conditions (LTCs), increase in prevalence and burden with ageing. Living with MLTCs reduces functional capacity and health-related quality of life, increases healthcare utilisation and costs, and increases all-cause mortality [1–3]. Adults living with pre-existing MLTCs are at increased risk of both developing severe coronavirus disease 2019 (COVID-19) after contracting severe acute respiratory syndrome coronavirus 2 (SARS-CoV-2) infection [4] and of persistent and more severe sequalae post-COVID-19 [5–7]. Specific underlying LTCs and MLTCs may exacerbate pathological mechanisms or reduce an individuals resilience against organ injury associated with COVID-19 and post-acute sequalae [8]. Long COVID (or “post COVID-19 condition” as defined by the World Health Organization (WHO) [9]) is a multisystemic condition encompassing a wide range of physical and psychosocial consequences with potentially compounding impacts for individuals with pre-existing MLTCs.

Individualised exercise-based (physical) rehabilitation has an established role in the management of a large number of LTCs [10]. In people living with MLTCs, exercise-based interventions have been reported to improve health-related quality of life and physical function, and reduce symptoms of anxiety and depression [11]. In clinical practice, the provision of generally single-condition-focused rehabilitation programmes and the heterogeneity of MLTCs [12] have implications for selection and tailoring of interventions for people with MLTCs. Other rehabilitation strategies include active mind–body movement interventions, *e.g.* yoga, Tai Chi and dance, where there is a growing body of evidence they improve health and wellbeing outcomes of people living with several specific LTCs [13–18].

In long COVID, multiple single-site, small randomised controlled trials of physical (exercise- and physical activity-based) rehabilitation interventions have demonstrated potential to improve functional exercise capacity and health-related quality of life for some adults with long COVID [19, 20]. Whilst largely positive, meta-analyses to date have been limited by small sample sizes and heterogeneity of both interventions and outcome measures [19–22].

A growing body of evidence has identified distinct symptom groups and phenotypes of long COVID [23]. Some of these phenomena, such as dysautonomia, postural orthostatic tachycardia syndrome (POTS), breathing pattern disorder and post-exertional symptom exacerbation (PESE), may challenge or limit physical rehabilitation similar to myalgic encephalomyelitis/chronic fatigue syndrome. Furthermore, health inequalities are associated with both MLTCs and long COVID, *e.g.* socioeconomic deprivation is associated with a higher prevalence of MLTCs [12] and incidence of long COVID after SARS-CoV-2-infection [5, 24]. Health and digital literacy, and practical aspects such as transport costs, may affect engagement, uptake and completion of rehabilitation programmes for long COVID. There are therefore likely to be additional rehabilitation needs and challenges for people with both pre-existing MLTCs and long COVID.

Whether physical rehabilitation interventions for long COVID are suitable, accessible, acceptable and effective in improving the health and wellbeing of individuals with pre-existing MLTCs is currently unclear. The presence of MLTCs may influence the selection and tailoring of appropriate rehabilitation interventions, and therefore reporting of pre-existing MLTCs is required for the evaluation of long COVID rehabilitation interventions. However, in available studies the results of rehabilitation interventions for people living with long COVID did not appear to be commonly reported according to individuals with and without pre-existing LTCs, and therefore a systematic review of the impact of pre-existing MLTCs is not currently possible. We therefore need to understand how pre-existing MLTCs are reported in the available evidence describing physical rehabilitation interventions for adults living with long COVID, and where MLTCs are reported, to understand the characteristics of the interventions and outcomes. This scoping review therefore aims to systematically map the extent and nature of reporting of pre-existing MLTCs within the available evidence for physical rehabilitation interventions for adults living with long COVID, and to describe the characteristics of physical rehabilitation interventions used in adults with both pre-existing LTCs and long COVID. Any gaps in the literature will be identified to inform recommendations for reporting of pre-existing MLTCs in long COVID rehabilitation research.

## Methods

The search methods and results are reported in accordance with the latest Joanna Briggs Institute (JBI) guidance for scoping reviews [25] and the PRISMA-ScR (Preferred Reporting Items for Systematic reviews and Meta-Analyses extension for Scoping Reviews) checklist [26] (see supplementary material).

### Protocol and registration

The protocol was registered on the Open Science Framework [27].

### Eligibility criteria

#### Participants

Eligibility criteria included adults (aged ≥18 years) living with “long COVID” (or “post COVID-19 condition” or “post COVID-19 syndrome”) defined in accordance with the WHO clinical case definition [9]:

“… occurs in individuals with a history of probable or confirmed SARS-CoV-2 infection, usually 3 months from the onset of COVID-19 with symptoms that last for at least 2 months and cannot be explained by an alternative diagnosis. Common symptoms include fatigue, shortness of breath, cognitive dysfunction but also others which generally have an impact on everyday functioning”.

#### Concept

Rehabilitation interventions including a physical activity component defined as repetitive whole-body movement [28] including but not limited to structured exercise (*e.g.* walking and Tai Chi) were considered for this review. The intervention could be physical only or physical in addition to psychosocial, educational, pharmaceutical and/or other adjunct interventions. The intervention could be supervised (including remote delivery, *e.g. via* video conferencing) or unsupervised, in any setting (*e.g.* hospital, community and home-based) and of any duration. Intervention timing is reported according to the length of time post-SARS-CoV-2 infection where available.

#### Context

No contextual limitations (*e.g.* geographical location or care setting) were applied in this review.

#### Types of evidence sources

Study designs that examine interventions or intervention content (*e.g.* controlled trials, crossover/cluster designs, cohort studies and qualitative studies relating to interventions), systematic literature reviews (*e.g.* systematic reviews and scoping reviews) and clinical guidelines were included. Case series and case reports were excluded. Relevant opinion-based pieces (*e.g.* editorials and letters) were used for reference checking only. Clinical guidelines specifically focused on long COVID rehabilitation were included. No other restrictions were placed in terms of study design or the form of article (*e.g.* quantitative or qualitative, or published or unpublished (where available)). Translation software was used to translate non-English articles to English.

### Information sources

MEDLINE, CINAHL, Scopus, APA PsycInfo, medRxiv (preprint server), OpenGrey and MedNar (grey literature databases) were searched, and manual searching of relevant grey literature (for relevant reports, doctoral theses and clinical guidelines) was conducted. Reference lists of included articles were checked for possible additional articles.

Databases were searched from 1 January 2020 (following identification of the SARS-CoV-2 virus) to 11 July 2023.

### Search strategy

The key concepts of long COVID (informed by a relevant recent systematic review [29]) and physical rehabilitation interventions were used to frame the search strategy (see supplementary material).

Following removal of duplicates, screening of titles/abstracts and full-text articles for eligibility was conducted by two independent authors (L. Gardiner and either H.M.L. Young or H. Drover or E. Morgan-Selvaratnam). Screening took place in Covidence (www.covidence.org), a web-based collaboration software platform that streamlines the production of systematic and other literature reviews. Disagreements between reviewers were resolved through discussion (L. Gardiner, H.M.L. Young, H. Drover and E. Morgan-Selvaratnam).

### Data extraction

Data were extracted from articles included in the scoping review by two independent reviewers (L. Gardiner and either H.M.L. Young or H. Drover or E. Morgan-Selvaratnam) using a data extraction tool developed within Covidence (see supplementary material) informed by the research questions, the JBI Manual for Evidence Synthesis [25], the TIDieR (Template for Intervention Description and Replication) checklist [30] and the CERT (Consensus on Exercise Reporting Template) [31].

Country of article origin and associated income classification was reported in accordance with the latest World Bank classification [32].

Where pre-existing LTCs were reported, the tool facilitated the collection of data regarding how pre-existing LTCs are described (number, type, specific named conditions and severity, and weighted measure) and whether individuals with pre-existing LTCs were analysed or considered separately from those without. Further to characteristics of associated long COVID physical rehabilitation interventions (including type of physical activity, timing post-SARS-CoV-2 infection, and mode and setting of intervention delivery and adjunctive interventions), domains of outcome measures reported (*e.g.* exercise capacity and health-related quality of life) were also extracted. Three reviewers (L. Gardiner, H.M.L. Young and H. Drover) each piloted the data extraction tool on three articles before wider use. Refinements included adding “reporting of severity of pre-existing LTCs” and “number of long COVID participants” (additional to “total number of participants”) to cater for studies that included individuals with acute/post-acute COVID-19 (as well as long COVID). Disagreements between reviewers were resolved through discussion.

Owing to apparent inconsistency in the use of pre-existing LTC-related terminologies and associated definitions, a decision was made to adopt an inclusive approach (*i.e.* include any terminology that may be considered pre-existing LTCs, such as “comorbidities” and “coexisting disease”). Due to limited ability to discern individuals with two or more pre-existing LTCs (from those with a single LTC), a further decision was made to report on all applicable studies that reported or referred to pre-existing LTCs.

### Data analysis and presentation

Microsoft Excel (www.microsoft.com) was used to map the extracted data to highlight the extent and nature of existing evidence and gaps in the literature in accordance with the research question. Extracted data were organised according to associated research questions. Characteristics of eligible articles were summarised using simple descriptive statistics (k=number of articles and n=number of participants). A results-based convergent synthesis design was employed [33] informed by the research questions. Quantitative and qualitative data were analysed separately and presented using tables, figures and narrative summary (organised by research questions). A mixed methods joint display [34] was used to present integrated findings. Data visualisation software Flourish (https://flourish.studio) was used to create figures.

## Results

**FIGURE 1:**
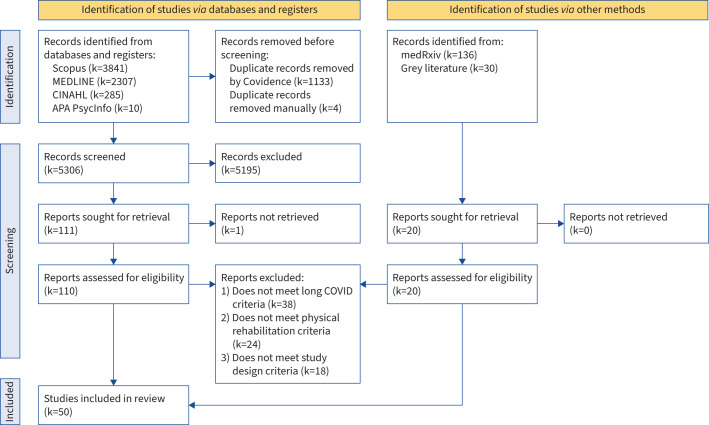
PRISMA (Preferred Reporting Items for Systematic Reviews and Meta-Analyses) flow diagram summarising the identification of articles [85].

Following removal of duplicates, the search strategy yielded a total of 5326 titles and abstracts. Following screening, 130 full texts were assessed for eligibility, of which 50 eligible articles were identified [35–84] (figure 1 [85]).

### Characteristics of articles

A summary of the characteristics of the 50 eligible articles is provided in the supplementary material. All articles were published between 2021 and 2023, and the majority (k=41 (82%)) originated from high-income countries. The number of participants for individual studies ranged from n=5 to n=8724. The eligible articles included eight systematic literature reviews including two protocols, 11 randomised control trials including five protocols, two randomised feasibility studies, seven non-randomised experimental studies including one protocol, 15 cohort studies, two case–control studies, two mixed methods studies, one care pathway development and two consensus statements.

### Reporting of pre-existing MLTCs

Of the 50 eligible articles, 25 (50%) [36, 37, 39, 42, 43, 45–47, 49, 51, 53–55, 58–60, 62, 70–72, 75, 76, 78, 80, 83] reported or referred to pre-existing LTCs and five (10%) [40, 44, 65, 74, 79] excluded several listed pre-existing LTCs within their eligibility criteria (see supplementary material). One study (4%) [43] reported single pre-existing LTCs but excluded individuals with MLTCs. The majority referred to pre-existing LTCs as “comorbidities” (k=20/25 (80%)). Four protocols [39, 47, 62, 83] referenced their intention to report past medical history, and one consensus statement [45] detailed consideration of pre-existing LTCs as part of comprehensive and holistic assessment for COVID-19 rehabilitation (including long COVID) within service delivery standards (see supplementary material for details).

**FIGURE 2:**
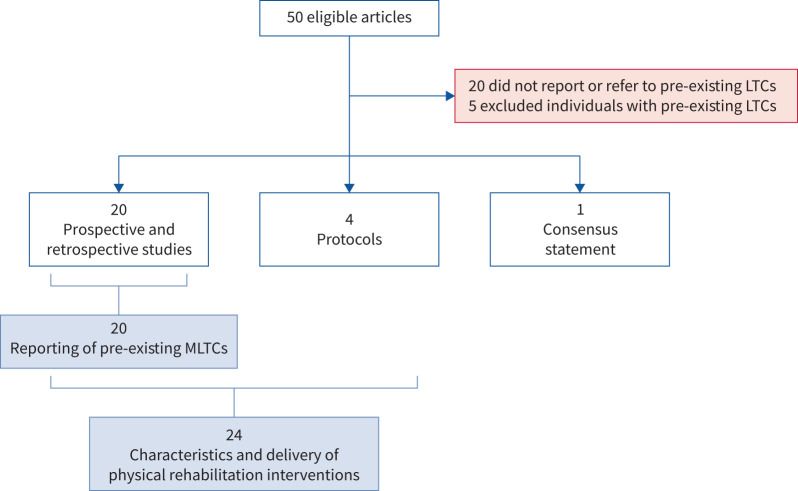
Flow diagram summarising applicable studies according to topic. LTCs: long-term conditions; MLTCs: multiple long-term conditions.

**FIGURE 3:**
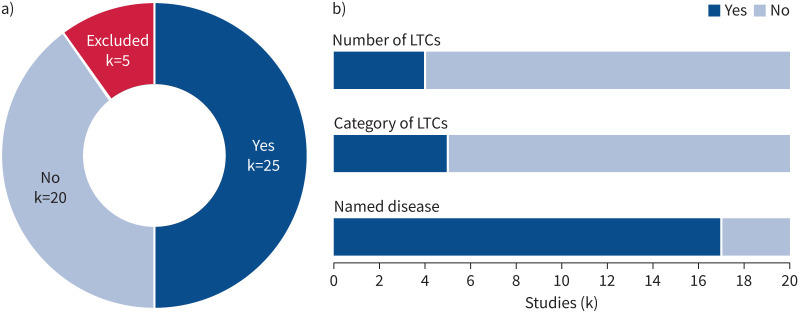
Reporting of pre-existing long-term conditions (LTCs): a) reporting or reference to pre-existing LTCs (k=50) and b) characteristics of pre-existing LTCs (k=20 applicable studies).

**FIGURE 4:**
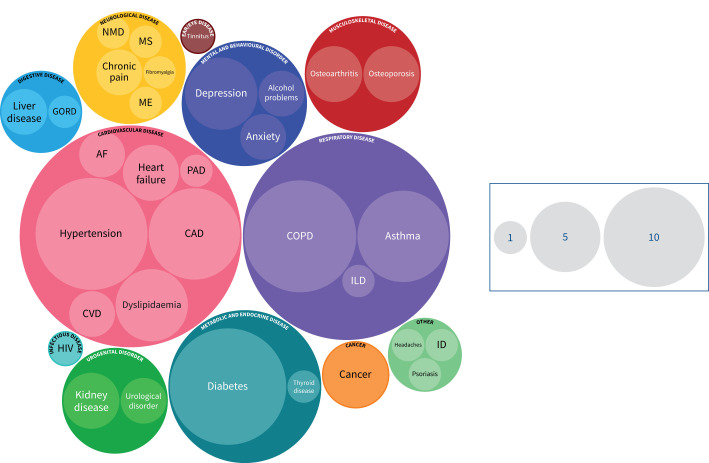
Bubble map of reporting of specific named pre-existing long-term conditions (LTCs) (k=20). Bubbles: body system (informed by International Classification of Diseases, 11th Revision). Bubble size: number of studies reporting the named LTC. AF: atrial fibrillation; CAD: coronary artery disease; CVD: cerebrovascular disease; GORD: gastro-oesophageal reflux disease; ID: immunological disorder; ILD: interstitial lung disease; ME: myalgic encephalomyelitis; MS: multiple sclerosis; NMD: neuromuscular disease; PAD: peripheral artery disease.

Of 20 applicable studies [36, 37, 42, 43, 46, 49, 51, 53–55, 58–60, 70–72, 75, 76, 78, 80] (excluding protocols [39, 47, 62, 83] and consensus statement [45]) (figure 2) that reported pre-existing LTCs, four (20%) [51, 53, 58, 78] reported the number of LTCs, enabling the differentiation of individuals with pre-existing MLTCs (figure 3 and table 1). The type or category of LTCs (*e.g.* respiratory, cardiovascular or metabolic disease) was reported by five out of 20 (25%) studies [51, 54, 59, 60, 72] and specific named LTCs (*e.g.* COPD, hypertension and diabetes mellitus) by 17 out of 20 (85%) studies [36, 37, 42, 43, 46, 49, 51, 53, 55, 59, 60, 70–72, 75, 76, 80]. The most commonly reported LTCs were diabetes (k=13/20 (65%)), COPD (k=12/20 (60%)) and hypertension (k=12/20 (60%)) (table 1 and figure 4). No articles reported or referred to severity of pre-existing LTCs. One study [76] referenced use of a weighted measure of pre-existing LTCs (Self-Administered Comorbidity Questionnaire (German) [86]) although this was used to report the frequency of specific named LTCs only. Three studies [53, 54, 58] analysed outcomes of individuals with pre-existing LTCs separately to those without. One study [58] reported outcomes of individuals with MLTCs separately to those without.

**TABLE 1:** Reporting of pre-existing long-term conditions (LTCs) in applicable studies (k=20)

First author, year [ref.] Country Study design (participants)	How pre-existing LTCs are reported	Severity of pre-existing LTCs reported?	Able to identify individuals with MLTCs?	Use or reference to weighted measures of MLTCs reported?	Outcomes of individuals with pre-existing LTCs analysed separately to those without	Participants with pre-existing LTCs/MLTCs (where available)^#^	
**Altmann, 2023 [36]** **Germany** **Cohort (n=42)**	Specific named	No	No	No	No	COPD or asthma	10/42 (23.8)
**Barbara, 2022 [37]** **Italy** **Cohort (n=50)**	Specific named	No	No	No	No	Hypertension	19/50 (38.0)
						Diabetes	4/50 (8.0)
						Dyslipidaemia	19/50 (38.0)
						Chronic kidney disease	5/50 (10.0)
						Heart failure	9/50 (16.4)
						Previous myocardial infarction	6/50 (12.0)
						COPD	7/50 (14.0)
**Brough, 2022 [42]** **UK** **Cohort (n=22)**	Specific named	No	No	No	No	NA	NA
**Calvo-Paniagua, 2022 [43]** **Spain** **Non-randomised trial (n=68)**	Specific named	No	Excluded	No	No	Arterial hypertension	1/68 (1.5)
						Diabetes	6/68 (8.8)
						Obesity	34/68 (50.0)
						COPD	0/68 (0.0)
						Asthma	8/68 (11.8)
**Compagno, 2022 [46]** **Italy** **Cohort (n=30)**	Specific named	No	No	No	No	One or more LTCs	22/30 (73.3)
**Oliveira, 2023 [49]** **Brazil** **RCT (n=59)**	Specific named	No	No	No	No	Hypertension	26/59 (44.1)
						Heart disease	8/59 (13.6)
						Diabetes mellitus	6/59 (10.6)
						Other	19/59 (31.7)
**Estebanez-Pérez, 2022 [51]** ^¶^ **Spain** **Non-randomised trial (n=32)**	Number, type, specific named	No	Yes	No	No	One or more LTCs	28/32 (87.5)
						Two or more LTCs (MLTCs)	23/32 (71.9)
**Fowler-Davis, 2021 [53]** ^¶^ **UK** **Mixed methods (n=10)**	Number, specific named	No	Yes	No	Yes Qualitative case descriptions	One or more LTCs	5/10 (50.0)
						Two or more LTCs (MLTCs)	3/10 (30.0)
**Frisk, 2023 [54]** **Norway** **Non-randomised trial (n=78)**	Type	No	No	No	Yes Outcomes reported for participants with no, previous and ongoing psychiatric illness	Previous or ongoing psychiatric illness	36/78 (46.2)
**Grishechkina, 2023 [55]** **Russia** **Cohort (n=113)**	Specific named	No	No	No	No	COPD	5/113 (4.4)
						Arterial hypertension	42/113 (37.2)
						Coronary heart disease	8/113 (7.1)
						Diabetes mellitus	13/113 (11.5)
						Obesity	14/113 (12.4)
**Hentschel, 2022 [58]** ^¶^ **USA** **Case–control (n=8724)**	Number	No	Yes	No	Yes Long COVID symptoms reported across number of comorbidities (0, 1–2, 3–4, 5–6, ≥7)	One or more LTCs	5304/8724 (60.8)
						One or two LTCs	2041/8724 (23.4)
						Three or more LTCs	3263/8724 (37.4)
**Jimeno-Almazán, 2022 [59]** **Spain** **RCT (n=39)**	Type, specific named	No	No	No	No	Hypertension	1/39 (2.6)
						Diabetes	1/39 (2.6)
						Asthma	5/39 (12.8)
						Structural heart disease	3/39 (7.7)
						Cerebrovascular disease	1/39 (2.6)
						Psychiatric conditions	11/39 (28.2)
**Jimeno-Almazán, 2023 [60]** **Spain** **RCT (n=80)**	Type, specific named	No	No	No	No	One or more LTCs	53/80 (66.3)
**Nopp, 2022 [70]** **Austria** **Cohort (n=58)**	Specific named	No	No	No	No	COPD	1/58 (1.7)
						Emphysema	2/58 (3.4)
						Asthma	11/58 (19.0)
						Coronary artery disease	3/58 (5.2)
						Arterial hypertension	13/58 (22.4)
						Diabetes mellitus	6/58 (10.3)
						Atrial fibrillation	1/58 (1.7)
						Hyperlipidaemia	18/58 (31.0)
						Hyperuricaemia	5/58 (8.6)
						Thyroid disease	5/58 (8.6)
						Renal disease	0/58 (0.0)
						Liver disease	3/58 (5.2)
**Ostrowska, 2023 [71]** **Poland** **Cohort (n=97)**	Specific named	No	No	No	No	Hypertension	45/97 (46.4)
						Hyperlipidaemia	24/97 (27.7)
						Coronary artery disease	18/97 (18.6)
						Heart failure	6/97 (6.2)
						COPD	12/97 (12.4)
**Parker, 2023 [72]** **UK** **Cohort (n=31)**	Type, specific named	No	No	No	No	Hypertension	5/31 (16.1)
						Diabetes	4/31 (12.9)
						Respiratory conditions	3/31 (9.7)
						Anxiety	5/31 (16.1)
						Depression	4/31 (12.9)
						Cardiovascular conditions	4/31 (12.9)
**Romanet, 2023 [75]** **France** **RCT (n=60)**	Specific named	No	No	No	No	Alcoholism	4/60 (6.7)
						COPD	5/60 (8.3)
						Cardiac insufficiency	2/60 (3.3)
						Ischaemic cardiopathy	3/60 (5.0)
						Occlusive arteriopathy	1/60 (1.7)
						Atrial fibrillation	3/60 (5.0)
						Cirrhosis	1/60 (1.7)
						Diabetes	22/60 (37.0)
						Cancer	3/60 (5.0)
						HIV	1/60 (1.7)
**Rutsch, 2023 [76]** **Germany** **Mixed methods (n=221)**	Specific named	No	No	Yes SCQ-D [86]	No	Hypertension	88/221 (39.8)
						Elevated blood lipids	88/221 (39.8)
						Osteoarthritis	70/221 (31.7)
						Gastric mucosal inflammation	64/221 (29.0)
						Bronchial asthma	62/221 (28.1)
						Depression	52/221 (23.5)
						Kidney disease	26/221 (11.8)
						Chronic bronchitis	22/221 (10.0)
						Diabetes mellitus	19/221 (8.6)
						Inflammatory joint diseases	18/221 (8.1)
						Circulatory disorders	13/221 (5.9)
						Osteoporosis	9/221 (4.1)
						Cancer	9/221 (4.1)
						Elevated blood lipids	88/221 (39.8)
**Smith, 2023 [78]** ^¶^ **UK** **Cohort (n=601)**	Number	No	Yes	No	No	Comorbidities	2.9±1.7
**Szarvas, 2023 [80]** **Hungary** **Non-randomised trial (n=68)**	Specific named	No	No	No	No	One or more LTCs	65/68 (95.6)

MLTCs: multiple long-term conditions; NA: not available; RCT: randomised controlled trial; SCQ-D: Self-Administered Comorbidity Questionnaire (German). ^#^: n/N (%) or mean±sd; ^¶^: studies that reported number of pre-existing LTCs, enabling the differentiation of individuals with MLTCs.

### Characteristics and delivery of physical rehabilitation interventions

A summary of characteristics and delivery of physical rehabilitation interventions in 24 applicable studies (that reported the presence of pre-existing LTCs) [36, 37, 39, 42, 43, 46, 47, 49, 51, 53–55, 58–60, 62, 70–72, 75, 76, 78, 80, 83] (excluding consensus statement [45]) (figure 2) is provided in the supplementary material. The most commonly reported type was combined aerobic and strengthening exercise (k=17 (70.8%)); one study [42] reported the use of active mind–body interventions (QiGong and seated yoga) in addition to functional exercise. Most interventions had a face-to-face supervised component (k=19 (79%)) and were based in an outpatient setting (k=13 (54%)). Four studies [51, 53, 72, 78] reported home-based telerehabilitation interventions (16.7%) and two studies [53, 72] reported unsupervised interventions (8.3%). Most studies reported or referenced tailoring to individuals' needs (k=20 (83%)), three specifically reported tailoring according to pre- or coexisting LTCs [36, 53, 80], but none of the studies provided any description of specific approaches according to LTCs (supplementary material). Physical rehabilitation was delivered with one or more adjunctive interventions in 15 studies (63%); breathing exercises (k=10) and education (k=9) were the most commonly reported types. A summary of timing of intervention delivery post-SARS-CoV-2 infection (reported within nine studies [36, 43, 46, 54, 55, 60, 70, 72, 78]) is provided in the supplementary material. The only consensus statement identified [45] referred to the need for individualisation of rehabilitation due to differences in individuals’ abilities and needs with specific reference to the presence of pre-existing LTCs.

### Outcome measure domains

The most commonly reported outcome measure domains within 24 applicable articles (excluding consensus statement [45]) was health-related quality of life (k=18 (75%)) (see supplementary material). Exercise capacity, functional ability and disability were reported by 17 studies (70.8%). Least frequently reported were frailty, mortality and hospitalisation (all k=1 (4%)). Reported outcome measures relating to long COVID symptoms included fatigue (k=8 (33%)), anxiety and depression (k=7 (29%)), dyspnoea (k=5 (21%)) and cognitive impairment (k=2 (8%)). Work-related outcomes (*e.g.* work ability and sick days) were reported by six studies (25%).

## Discussion

To the best of the authors’ knowledge, this is the first scoping review to map the reporting of pre-existing MLTCs in physical rehabilitation intervention studies for long COVID. Our scoping review has identified limited and inconsistent reporting of the presence and impact of pre-existing MLTCs within the long COVID physical rehabilitation literature. Individuals with MLTCs were distinguishable from those without in only 20% of the studies that reported pre-existing LTCs and only one study reported intervention outcomes separately for individuals with MLTCs compared to those without. Physical rehabilitation interventions investigated most commonly consisted of aerobic and strengthening exercise and were delivered in an outpatient setting. The most commonly reported outcome domains were health-related quality of life, exercise capacity, functional ability and disability, in keeping with other rehabilitation programmes for LTCs [87].

With the global prevalence of adults (≥18 years) with MLTCs estimated at 37.2% (95% CI 34.9–39.4%) [88] and with people with MLTCs being at higher risk of long COVID, most studies would be expected to involve participants with MLTCs (unless explicitly excluded), yet 40% of the studies in the current review made no reference to individuals with pre-existing LTCs. The majority of studies that reported pre-existing LTCs (85%) reported frequency of single named LTCs, providing some characterisation relevant to the presence of MLTCs. No trials or protocols identified by this review specifically sought to recruit individuals with one or more pre-existing LTCs and no justification was identified for excluding individuals with pre-existing LTCs within the relevant studies. Whilst there may be valid justification in certain circumstances, excluding individuals with pre-existing LTCs in trials of long COVID rehabilitation may result in interventions not being acceptable and effective for many of the target population [89]. In clinical practice, individuals with pre-existing MLTCs may require adaptations to assessment and intervention delivery, including safety, exercise prescription and self-management considerations as part of a personalised, holistic approach [45, 90]. It is therefore important to be able to understand the results of rehabilitation studies in long COVID between those with and without LTCs. Whilst some studies (15%) compared outcomes of individuals with and without pre-existing LTCs, demonstrating recognition of potential impact on care needs and outcomes, none were randomised controlled trials. The challenge of designing and evaluating MLTC-specific intervention trials (compared to single LTCs) has also been recognised [91]. Variation in terminologies used to describe MLTCs compounds limitations in MLTCs evidence synthesis [91, 92] and most articles identified by this review referred to pre-existing MLTCs as “comorbidities”. However, “MLTCs” is recognised as a preferred term by patient and the public [93], and as a distinct concept from terms that infer coexistence of disease secondary to an index condition [94]. In the current review, there were notable inconsistencies in the breadth of LTCs and associated types described, in keeping with inconsistent definitions and reporting of MLTCs within broader MLTCs research and relevant guidelines [12, 92, 95]. An international Delphi consensus study (professional and public panels) previously sought to provide guidance on defining and measuring MLTCs in research, and defined MLTCs as two or more LTCs and described 59 LTCs to consistently include [96]. The LTCs were selected as they “significantly reduce quality of life, significantly worsen mental health, significantly increase risk of death, cause frailty, cause physical disability or significantly increase treatment burden” [96].

Some researchers have proposed the concept of “complex” or “severe” MLTCs [12, 97], but a consensus definition is yet to be established [12, 96, 97]. The severity of pre-existing LTCs was not reported by any study in the current review. There is increasing understanding about how MLTCs cluster together [91, 98] which may also inform future reporting recommendations for MLTCs. Reporting of long COVID phenotypes, which may be influenced by the presence of pre-existing MLTCs [8], is also of particular relevance to identifying suitable rehabilitation interventions for this population. One study [76] in the current review used a weighted measure of pre-existing LTCs. Weighted indices of MLTCs such as the Cambridge Multimorbidity Score [99] introduce a weighting attributable to included LTCs based on severity and/or impact [12]. Whilst simple condition count does not account for the nature or severity of LTCs, a systematic review of MLTCs measures reported it to be the only measure associated with three core MLTCs outcomes (quality of life, mental health and mortality) [100]. Authors of relevant reviews [95, 100, 101] have therefore recommended researchers align their choice of MLTCs measure with the aims and outcomes of the study.

Supervised aerobic and strengthening exercise was the most common type of intervention used in the applicable studies of long COVID physical rehabilitation interventions, perhaps unsurprisingly given the established role in the management of a large number of LTCs [10]. The delivery of individualised aerobic and strengthening exercise as part of evidence-informed pulmonary and cardiac rehabilitation is reliant on individualised prescription (based on a validated exercise test) of traditional forms of exercise such as walking, cycling and weighted exercises [102–104]. Trials of long COVID physical rehabilitation to date have limited reporting of phenotypic characteristics that are pertinent to appropriately individualised and symptom-titrated exercise prescription [105], such as PESE, POTS, breathing pattern disorder and fatigue. Adults living with pre-existing MLTCs and long COVID may experience compounded impacts of long COVID requiring further considerations for the provision of appropriate exercise prescription. Timing of physical rehabilitation delivery (post-SARS-CoV-2 infection), which was inconsistently reported (k=9 (45.0%)) and variable (∼3–17 months post-infection), may also have relevance to the suitability and efficacy of the intervention.

In keeping with long-standing LTC rehabilitation programmes [102, 103], physical rehabilitation interventions were commonly delivered alongside adjunctive interventions (*e.g.* breathing exercises and education). Reporting of pre-existing MLTCs in broader rehabilitation intervention trials may support the identification and/or appropriate adaptations of adjunctive interventions that are suitable and effective in improving health and wellbeing outcomes in this population.

Whilst the majority of applicable studies reported outcome domains pertinent to the aims of physical rehabilitation interventions (including health-related quality of life and exercise capacity), relatively few reported outcome measures relating to long COVID symptoms (*e.g.* fatigue (k=8 (33%)) and cognitive impairment (k=2 (8%))). A standardised approach to outcome reporting in long COVID rehabilitation research is needed to ensure that critical outcomes (relevant to the breadth of the long COVID population) are measured and reported consistently, and to reduce heterogeneity that may otherwise limit evidence synthesis [106]. A core outcome set developed for MLTCs research included domains of treatment burden, shared decision making and prioritisation that may be pertinent to individuals with pre-existing MLTCs and long COVID [107].

Our scoping review has highlighted a variety of descriptions of MLTCs and therefore there is a need for consistency/standardisation of reporting. There is ongoing debate about how to meaningfully describe MLTCs that relate to phenotypes and outcomes [12, 92, 97]. For rehabilitation, ultimately it would be desirable to classify MLTCs according to phenotypes that require specific interventions. Possible MLTCs characteristics for reporting may include singled named LTCs, index condition, categories of LTCs (or body systems affected), severity of LTCs, phenotypic characteristics (*e.g.* breathlessness), clusters (informed by relevant evidence) and weighted measures of MLTCs. Authors should also report where specific LTCs have been excluded with justification.

### Strengths and limitations

A strength of this review is the robust systematic methodology including a wide and comprehensive search strategy to optimise inclusivity of literature relevant to those experiencing long-term sequalae of SARS-CoV-2 infection. However, varied use of post-COVID-19-related terminologies and inconsistent reporting of definitions (particularly in articles predating the WHO clinical case definition of “long COVID” [9]) may have led to exclusion of potentially relevant articles.

An inherent limitation of this review was MLTCs being considered a “single entity” as it is important to acknowledge the heterogeneity of MLTCs and implications for tailoring physical rehabilitation interventions. Also, how the presence of pre-existing LTCs was reported (*i.e.* from medical records or self-reported) was not within the scope of this review; however, it is an important consideration for the accurate reporting of MLTCs. Mapping the reporting of phenotypic characteristics of long COVID was also beyond the scope of this review but is pertinent to the evaluation of rehabilitation interventions in this population.

## Conclusions

We report limited and inconsistent reporting of the presence and impact of MLTCs in studies of physical rehabilitation interventions for people with long COVID. We therefore recommend consistent and appropriately detailed reporting of pre-existing MLTCs in research to enable appropriate adaptation and evaluation of rehabilitation.

**Points for clinical practice and questions for future research**

- Reporting of pre-existing MLTCs is required to enable adaptation and evaluation of long COVID physical rehabilitation interventions.
- Further work is required to inform comprehensive recommendations for the reporting of pre-existing MLTCs within long COVID rehabilitation studies.

## Supplementary material

10.1183/16000617.0123-2024.Supp1 **Please note:** supplementary material is not edited by the Editorial Office, and is uploaded as it has been supplied by the author.
Supplementary material ERR-0123-2024.SUPPLEMENT
