# Supplementary material for: Reporting of pre-existing multiple long-term conditions in physical rehabilitation for long COVID: a scoping review
Source: Eur Respir Rev. 2024 Nov 27;33(174):240123. doi: 10.1183/16000617.0123-2024 (PMC11600128; doi:10.1183/16000617.0123-2024)

PRISMA-ScR Checklist [26]

SECTION	ITEM	PRISMA-ScR CHECKLIST ITEM	REPORTED ON PAGE #
**TITLE**			
Title	1	Identify the report as a scoping review.	1
**ABSTRACT**			
Structured summary	2	Provide a structured summary that includes (as applicable): background, objectives, eligibility criteria, sources of evidence, charting methods, results, and conclusions that relate to the review questions and objectives.	2
**INTRODUCTION**			
Rationale	3	Describe the rationale for the review in the context of what is already known. Explain why the review questions/objectives lend themselves to a scoping review approach.	3-4
Objectives	4	Provide an explicit statement of the questions and objectives being addressed with reference to their key elements (e.g., population or participants, concepts, and context) or other relevant key elements used to conceptualize the review questions and/or objectives.	3-4
**METHODS**			
Protocol and registration	5	Indicate whether a review protocol exists; state if and where it can be accessed (e.g., a Web address); and if available, provide registration information, including the registration number.	5
Eligibility criteria	6	Specify characteristics of the sources of evidence used as eligibility criteria (e.g., years considered, language, and publication status), and provide a rationale.	5-6
Information sources*	7	Describe all information sources in the search (e.g., databases with dates of coverage and contact with authors to identify additional sources), as well as the date the most recent search was executed.	6-7
Search	8	Present the full electronic search strategy for at least 1 database, including any limits used, such that it could be repeated.	Online supplementary material pp. 3-5
Selection of sources of evidence†	9	State the process for selecting sources of evidence (i.e., screening and eligibility) included in the scoping review.	7
Data charting process‡	10	Describe the methods of charting data from the included sources of evidence (e.g., calibrated forms or forms that have been tested by the team before their use, and whether data charting was done independently or in duplicate) and any processes for obtaining and confirming data from investigators.	7-8
Data items	11	List and define all variables for which data were sought and any assumptions and simplifications made.	Online supplementary material pp.6-8
Critical appraisal of individual sources of evidence§	12	If done, provide a rationale for conducting a critical appraisal of included sources of evidence; describe the methods used and how this information was used in any data synthesis (if appropriate).	Not applicable

SECTION	ITEM	PRISMA-ScR CHECKLIST ITEM	REPORTED ON PAGE #
Synthesis of results	13	Describe the methods of handling and summarizing the data that were charted.	8
**RESULTS**			
Selection of sources of evidence	14	Give numbers of sources of evidence screened, assessed for eligibility, and included in the review, with reasons for exclusions at each stage, ideally using a flow diagram.	9 and Figure 1
Characteristics of sources of evidence	15	For each source of evidence, present characteristics for which data were charted and provide the citations.	9 and online supplementary material pp.9-12
Critical appraisal within sources of evidence	16	If done, present data on critical appraisal of included sources of evidence (see item 12).	Not applicable
Results of individual sources of evidence	17	For each included source of evidence, present the relevant data that were charted that relate to the review questions and objectives.	11-13 and online supplementary material pp.9-22
Synthesis of results	18	Summarize and/or present the charting results as they relate to the review questions and objectives.	9, 14-15 and Figures 2-4
**DISCUSSION**			
Summary of evidence	19	Summarize the main results (including an overview of concepts, themes, and types of evidence available), link to the review questions and objectives, and consider the relevance to key groups.	15-18
Limitations	20	Discuss the limitations of the scoping review process.	19
Conclusions	21	Provide a general interpretation of the results with respect to the review questions and objectives, as well as potential implications and/or next steps.	19
**FUNDING**			
Funding	22	Describe sources of funding for the included sources of evidence, as well as sources of funding for the scoping review. Describe the role of the funders of the scoping review.	20

JBI = Joanna Briggs Institute; PRISMA-ScR = Preferred Reporting Items for Systematic reviews and Meta-Analyses extension for Scoping Reviews.

* Where *sources of evidence* (see second footnote) are compiled from, such as bibliographic databases, social media platforms, and Web sites.

† A more inclusive/heterogeneous term used to account for the different types of evidence or data sources (e.g., quantitative and/or qualitative research, expert opinion, and policy documents) that may be eligible in a scoping review as opposed to only studies. This is not to be confused with *information sources* (see first footnote).

‡ The frameworks by Arksey and O'Malley (6) and Levac and colleagues (7) and the JBI guidance (4, 5) refer to the process of data extraction in a scoping review as data charting.

§ The process of systematically examining research evidence to assess its validity, results, and relevance before using it to inform a decision. This term is used for items 12 and 19 instead of "risk of bias" (which is more applicable to systematic reviews of interventions) to include and acknowledge the various sources of evidence that may be used in a scoping review (e.g., quantitative and/or qualitative research, expert opinion, and policy document).

Search strategies (informed by O’Mahoney et al. [29])

MEDLINE:

1 (long adj3 (covid* or ncov* or novel coronavirus or novel betacoronavirus or sars-ncov-2 or sars-cov-2)).tw,kw. 3913
2 (persist* adj5 (covid* or ncov* or novel coronavirus or novel betacoronavirus or sars-ncov-2 or sars-cov-2)).tw,kw. 2975
3 (chronic adj3 (covid* or ncov* or novel coronavirus or novel betacoronavirus or sars-ncov-2 or sars-cov-2)).tw,kw. 775
4 ((long term or long-term or longterm) adj3 effect* adj3 (covid* or ncov* or novel coronavirus or novel betacoronavirus or sars-ncov-2 or sars-cov-2)).tw,kw. 475
5 (sequela* adj3 (covid* or ncov* or novel coronavirus or novel betacoronavirus or sars-ncov-2 or sars-cov-2)).tw,kw. 1736
6 ((post acute or post-acute or postacute) adj3 (covid* or ncov* or novel coronavirus or novel betacoronavirus or sars-ncov-2 or sars-cov-2)).tw,kw. 1089
7 ((longhaul* or long haul* or long-haul*) adj3 (covid* or ncov* or novel coronavirus or novel betacoronavirus or sars-ncov-2 or sars-cov-2)).tw,kw. 188
8 ((post-covid or postcovid) adj syndrome).tw,kw. 356
9 exp Post-Acute COVID-19 Syndrome/ 2216
10 ((ongoing or on-going or on going) adj3 (covid* or ncov* or novel coronavirus or novel betacoronavirus or sars-ncov-2 or sars-cov-2)).tw,kw. 3617
11 ((symptomatic adj3 (covid* or ncov* or novel coronavirus or novel betacoronavirus or sars-ncov-2 or sars-cov-2)) and ((chronic* and longterm) or long term or long-term)).tw,kw. 111
12 ((chronic* or longterm or long term or long-term) adj3 (post covid or post-covid or postcovid)).tw,kw. 157
13 (survivor* adj3 (covid* or ncov* or novel coronavirus or novel betacoronavirus or sars-ncov-2 or sars-cov-2)).tw,kw. 1754
14 ((recover* or discharg* or follow* or prolong*) adj5 (covid* or ncov* or novel coronavirus or novel betacoronavirus or sars-ncov-2 or sars-cov-2)).tw,kw. 20405
15 1 or 2 or 3 or 4 or 5 or 6 or 7 or 8 or 9 or 10 or 11 or 12 or 13 or 14 31526
16 Rehab*.tw,kw. 219073
17 exercis*.tw,kw. 362390
18 "physical activit*".tw,kw. 152685
19 "aerobic training".tw,kw. 3232
20 "resist* training".tw,kw. 11530
21 walking.tw,kw. 89544
22 ?cycling.tw,kw. 78635
23 "mind-body movement therap*".tw,kw. 9
24 danc*.tw,kw. 9404
25 yoga*.tw,kw. 6747
26 "tai?chi".tw,kw. 599
27 "tai?ji".tw,kw. 139
28 "qi?gong".tw,kw. 1000
29 telerehab*.tw,kw. 1954
30 physiotherap*.tw,kw. 34373
31 "physical therap*".tw,kw. 32599
32 exp Rehabilitation/ 352832
33 exp Exercise/ 246446
34 exp Exercise Therapy/ 63325
35 exp Exercise Movement Techniques/ 10285

36 16 or 17 or 18 or 19 or 20 or 21 or 22 or 23 or 24 or 25 or 26 or 27 or 28 or 29 or 30 or 31 or 32 or 33 or 34 or 35 1122553
37 15 and 36 **2271**

CINAHL:

S14: S5 AND S13 **282**
S13: S6 OR S7 OR S8 OR S9 OR S10 OR S11 OR S12 416370
S12: MJ physiotherap* or "physical therap*" 43456
S11: MJ telerehab* 536
S10: (MM "Mind Body Techniques+") 30336
S9: (("mind body" or mind-body or mindbody) AND "movement therap*") OR (danc* or yoga or "tai chi" or "tai-chi" or "taichi" or "tai ji" or "tai-ji" or "taiji" or "qi gong" or "qi-gong" or "qigong") 23916
S8: (MM "Exercise+") 78709
S7: (MM "Rehabilitation+") 221353
S6: MJ (rehab* or exercis* or "physical activit*") OR MJ ("aerobic training" or "resist* training") OR MJ (walking or ?cycling) 237890
S5: S1 OR S2 OR S3 OR S4 2569
S4: MJ (survivor* or recover* or discharg* or follow* or prolong*) AND MJ (covid* or ncov* or novel coronavirus or novel betacoronavirus or sars-ncov-2 or sars-cov-2) 905
S3: ("post covid*" or post-covid* or postcovid*) AND (syndrome or condition) 332
S2: (MM "Post-Acute COVID-19 Syndrome") 630
S1: MJ ((long or persist* or chronic or "long term" or long-term or longterm or sequala* or "post acute" or post-acute or postacute or "long haul" or long-haul or longhaul or "on going" or on-going or ongoing)) AND MJ ((covid* or ncov* or novel coronavirus or novel betacoronavirus or sars-ncov-2 or sars-cov-2)) 1522

SCOPUS:

12: #5 AND #11 **3725**
11: #6 OR #7 OR #8 OR #9 OR #10 1660640
10: TITLE-ABS-KEY (physiotherap* OR "physical therap*") 155967
9: TITLE-ABS-KEY (telerehab*) 3673
8: TITLE-ABS-KEY (danc* OR yoga OR "tai chi" OR "tai-chi" OR "taichi" OR "tai ji" OR "tai-ji" OR "taiji" OR "qi gong" OR "qi-gong" OR "qigong") 69576
7: TITLE-ABS-KEY (("mind body" OR mind-body OR mindbody) AND "movement therap*") 91
6: TITLE-ABS-KEY((rehab* or exercis* or "physical activit*") OR ("aerobic training" or "resist* training") OR (walking or ?cycling)) 1513872
5: #2 OR #3 OR #4 65258
4: TITLE-ABS-KEY ((survivor* OR recover* OR discharg* OR follow* OR prolong*) AND (covid* OR ncov* OR novel AND coronavirus OR novel AND betacoronavirus OR sars-ncov-2 OR sars-cov-2)) 42058
3: TITLE-ABS-KEY (("post covid*" OR post-covid* OR postcovid*) AND (syndrome OR condition)) 3972
2: TITLE-ABS-KEY ((long OR persist* OR chronic OR "long term" OR long-term OR longterm OR sequala* OR "post acute" OR post-acute OR postacute OR "long haul" OR long-haul OR longhaul OR "on going" OR on-going OR ongoing) AND (covid* OR ncov* OR novel AND coronavirus OR novel AND betacoronavirus OR sars-ncov-2 OR sars-cov-2)) 32392

APA PsychINFO:

S14: (S6 OR S7 OR S8 OR S9 OR S10 OR S11 OR S12) AND (S5 AND S13) **10**
S13: S6 OR S7 OR S8 OR S9 OR S10 OR S11 OR S12 90877

S12: MJ physiotherap* or "physical therap*" 3221
S11: MJ telerehab* 183
S10: MJ (("mind body" or mind-body or mindbody) AND "movement therap*") OR MJ (danc* or yoga or "tai chi" or "tai-chi" or "taichi" or "tai ji" or "tai-ji" or "taiji" or "qi gong" or "qi-gong" or "qigong") 5709
S9: MM "Movement Therapy" 1297
S8: MM "Exercise" OR MM "Aerobic Exercise" OR MM "Weightlifting" OR MM "Yoga" 26068
S7: MM "Rehabilitation" OR MM "Physical Therapy" OR MM "Telerehabilitation" 21117
S6: MJ ((rehab* or exercis* or "physical activit*")) OR MJ (("aerobic training" or "resist* training")) OR MJ ((walking or ?cycling)) 82256
S5: S1 OR S2 OR S3 OR S4 416
S4: MJ ((survivor* or recover* or discharg* or follow* or prolong*)) AND MJ ((covid* or ncov* or novel coronavirus or novel betacoronavirus or sars-ncov-2 or sars-cov-2)) 202
S3: ("post covid*" or post-covid* or postcovid*) AND (syndrome or condition) 0
S2: MM "Post-COVID-19 Conditions" 10
S1: MJ ((long or persist* or chronic or "long term" or long-term or longterm or sequala* or "post acute" or post-acute or postacute or "long haul" or long-haul or longhaul or "on going" or on-going or ongoing)) AND MJ ((covid* or ncov* or novel coronavirus or novel betacoronavirus or sars-ncov-2 or sars-cov-2)) 208

Data extraction tool (informed by the JBI manual for evidence synthesis [25], Template for Intervention Description and Replication (TIDieR) checklist [30], and Consensus on Exercise Reporting Template (CERT) [31])

**General Information**	
Study ID	
Title	
Country in which study conducted	

**Characteristics of Included Studies**<br>*Methods*	
Aim of study	
Study design	☐ Randomised controlled trial<br>☐ Non-randomised experimental study<br>☐ Cohort study<br>☐ Case control study<br>☐ Systematic literature review<br>☐ Qualitative research<br>☐ Intervention development<br>☐ Consensus statement<br>☐ Clinical guideline<br>☐ Other<br>*Details:*
Study setting	☐ Hospital inpatient<br>☐ Hospital outpatient<br>☐ Primary care<br>☐ Community<br>☐ Other<br>*Details:*
Participants	
Long Covid description	
Time post SARS-CoV-2 infection reported?	☐ Yes ☐ No ☐ Unclear<br>*Details:*
Inclusion criteria	
Exclusion criteria	
Total number of consented participants	
Protected characteristics reported?	☐ Age<br>☐ Disability<br>☐ Gender reassignment<br>☐ Marriage and civil partnership<br>☐ Pregnancy and maternity<br>☐ Race or ethnicity<br>☐ Religion or belief<br>☐ Sex<br>☐ Sexual orientation

	Details:
Pre-existing LTC reported?	☐ Yes ☐ No ☐ Several listed LTC excluded<br>*Details:*
If pre-existing LTC reported, how?	☐ Number<br>☐ Type (e.g., respiratory, cardiovascular)<br>☐ Specific named (e.g., COPD, Diabetes)<br>☐ Other<br>☐ Not applicable<br>*Details:*
If pre-existing LTC reported, is severity reported?	☐ Yes ☐ No ☐ Not applicable<br>*Details:*
If pre-existing LTC are reported, are you able to identify MLTC (i.e., pts with 2 or more LTC)?	☐ Yes ☐ No ☐ Not applicable<br>*Details:*
If pre-existing LTC reported, any weighted measures of MLTC reported (e.g., Charlson Index, Cambridge Multimorbidity score)?	☐ Yes ☐ No ☐ Not applicable<br>*Details:*
Intervention and comparison	
Brief name of physical rehabilitation intervention *(name or phrase that describes the intervention)*	
Rationale/theory/goal of essential elements of intervention	
Procedure of physical rehabilitation intervention *(describe all activities involved and any equipment/materials used)*	
Who provided the intervention? *(describe expertise, background and any training provided)*	
Mode(s) of delivery	☐ Face-to-face ☐ Individual<br>☐ Remotely delivered ☐ Group<br>☐ Unsupervised<br>*Details:*
Study setting if any face-to-face element(s)	☐ Hospital inpatient ☐ Not applicable<br>☐ Hospital outpatient<br>☐ Primary care<br>☐ Community<br>☐ Other<br>*Details:*
Frequency, intensity, and duration of intervention	
Any tailoring to individuals' needs? *(what, why, when, how)*	
Any modifications during the course of the study? *(what, why, when, how)*	
Any details reported on planned or actual adherence and/or fidelity	
Comparator details *(what, why,*	

when, how for each comparator)	
Outcomes	
Outcome domains reported	☐ Exercise capacity<br>☐ Functional ability<br>☐ Health-related quality of life<br>☐ Physical activity<br>☐ Frailty<br>☐ Disability<br>☐ Mortality<br>☐ Hospitalisation<br>☐ Other<br>*Details:*
If pre-existing LTC reported, are outcomes reported separately (to those without)?	☐ Yes ☐ No ☐ Not applicable<br>*Details:*
Summary of main findings	

Characteristics of eligible articles (*k=50*)

Article and year	Country	Article type / study design	Setting	Brief name of intervention	Number of consented participants with LC	Reporting/ reference to pre-existing LTC	
						**Yes ✓no× excluded**	**Descriptive term(s) +/- qualitative data**
Albu et al., 2022 [35]	Spain	Cohort study	Outpatient	Multidisciplinary outpatient rehabilitation	40	×	NA
Altmann et al., 2023 [36]	Germany	Cohort study	Inpatient	Rehabilitation program	42	✓	*Term:* 'comorbidities'
Barbara et al., 2022 [37]	Italy	Cohort study	Outpatient	Exercise rehabilitation	50	✓	*Term:* described within patient characteristics
Bernal-Utrera et al., 2022 [38]	Spain	Systematic review	NA	Therapeutic exercise interventions through telerehabilitation	NA	×	NA
Besnier et al., 2022 [39]	Canada	RCT protocol	Outpatient	Cardiopulmonary rehabilitation	NA	✓ (*some excluded*)	*Terms:* 'previous diseases'; 'comorbidities'
Bouteleux et al., 2021 [40]	France	Cohort study	Outpatient	Respiratory rehabilitation	39	Excluded	NA
Brehon et al., 2022 [41]	Canada	Cohort study	Unclear	Occupational rehabilitation	81	×	NA
Brough et al., 2022 [42]	United Kingdom	Cohort study	Community site	Community project combining psychoeducation and mind-body complementary approaches	25	✓	*Term:* 'existing health conditions'
Calvo-Paniagua et al., 2022 [43]	Spain	Non-randomised trial	Primary care (telerehabilitation)	Tele-health primary care rehabilitation program	68	✓ *(MLTC excluded)*	*Term:* 'pre-existing comorbidities' *Exclusion term*: 'pluri-pathology' (referring to MLTC)
Certain Curi et al., 2022 [44]	Brazil	RCT protocol	Outpatient	Osteopathy and physiotherapy	NA	Excluded	NA
Chartered Society of Physiotherapy, 2021 [45]	United Kingdom	Consensus statement	NA	Rehabilitation - Physiotherapy services	NA	✓	*Terms*: 'comorbidities', 'underlying health conditions'<br><br>"*1.2 Assessment is holistic and includes consideration of risk, co-morbidities, prognosis..*" (pp.6)<br><br>"*People with COVID-19 often present with a wide range of clinical, physical, psychological (including cognition), emotional, cultural and social needs due to the virus and also related to other underlying health conditions. A holistic assessment should consider all of these needs..*" (pp.7)

							"*Each individual with a diagnosis of COVID-19 may have other health conditions and may have very different abilities and rehabilitation needs. The aim of symptom management is to optimise the person's clinical status and ability to undertake rehabilitation"* (pp.12)
Compagno et al., 2022 [46]	Italy	Cohort study	Outpatient	Exercise and psychological - based rehabilitation program	30	✓	*Term:* 'comorbidities'
Daynes et al., 2023 [47]	United Kingdom	RCT protocol	Outpatient and home-based	COVID rehabilitation	NA	✓	*Term:* 'past medical history'
Decary et al., 2022 [48]	Multi-national authors	Scoping review	NA	Rehabilitation care models	NA	×	NA
deOliveira et al., 2023 [49]	Brazil	RCT	Unclear	Multicomponent rehabilitation	59	✓	*Term:* 'comorbidities'
Estebanez-Pérez et al., 2023 [50]	Spain	Systematic review	NA	Digital physiotherapy practice	NA	×	NA
Estebanez-Pérez et al., 2022 [51]	Spain	Non-randomised trial	Home-based (telerehabilitation)	Digital physiotherapy intervention	32	✓	*Term:* 'comorbidities'
Fernandez-Lazaro et al., 2022 [52]	Spain	Narrative review	NA	Therapeutic Exercise	NA	×	NA
Fowler-Davis et al., 2021 [53]	United Kingdom	Mixed methods	Home-based (telerehabilitation)	Virtual multidisciplinary LC rehabilitation intervention	10	✓	*Term:* 'comorbidities', 'pre-existing' named LTC<br><br>*Participant 3: "He had several co-morbidities including psoriasis, arthritis, tinnitus and depression"* (pp.9)<br><br>*Participant 5: "She lived alone with nearby family and had pre-existing COPD."* (pp.9)
Frisk et al., 2023 [54]	Norway	Non-randomised trial	Outpatient	Micro-choice based rehabilitation	78	✓	*Term:* 'previous or ongoing illness'
Grishechkina et al., 2023 [55]	Russia	Cohort study	Spa setting	Multidisciplinary rehabilitative interventions	120	✓	*Term:* 'comorbidities'
Groenveld et al., 2022 [56]	Netherlands	Non-randomised trial	Home-based	Virtual reality exercises	48	×	NA
Hasenoehrl et al., 2022 [57]	Austria	Cohort study	Outpatient	Physical exercise	28	×	NA
Hentschel et al., 2022 [58]	United States	Case-control study	Outpatient	Outpatient rehabilitation services	8724	✓	*Term:* 'comorbidities'
Jimeno-Almazán et al., 2022 [59]	Spain	RCT	Outpatient	Supervised exercise intervention	39	✓	*Term:* 'comorbidities'

Jimeno-Almazán et al., 2023 [60]	Spain	RCT	Outpatient	Concurrent training, respiratory muscle exercise, and self-management recommendations	80	✓	*Term:* 'comorbidities'
Kabir et al., 2023 [61]	Bangladesh	Scoping review	NA	Physiotherapy interventions	NA	×	NA
Kupferschmitt et al., 2022a [62]	Germany	Non-randomised trial protocol	Outpatient	Specialised rehabilitation: pneumological, cardiological, psychosomatic, neurological	NA	✓	*Term:* 'comorbidities'
Kupferschmitt et al., 2022b [63]	Germany	Case-control study	Inpatient	Post-COVID inpatient rehabilitation	51	×	NA
Liu et al., 2022 [64]	China	Systematic review protocol	NA	Traditional Chinese exercises	NA	×	NA
Lobanov et al., 2022 [65]	Russia	Feasibility randomised trial	Outpatient	Aquatic exercises	29	Excluded	NA
Mammi et al., 2023 [66]	Italy	Cohort study	Outpatient	Outpatient rehabilitation program	31	×	NA
Manhas et al., 2022 [67]	Canada	Care pathway development	NA	Novel Care Rehabilitation Pathway for Post-COVID Conditions (LC)	NA	×	NA
Marques-Silva et al., 2022 [68]	Mexico	Non-randomised trial	Unclear	Therapeutic exercise program	5	×	NA
McGregor et al., 2021 [69]	United Kingdom	RCT protocol	Home-based (telerehabilitation)	Rehabilitation exercise and psychological support	NA	×	NA
Nopp et al., 2022 [70]	Austria	Cohort study	Outpatient	Outpatient pulmonary rehabilitation	58	✓	*Term:* 'comorbidities'
Ostrowska et al., 2023 [71]	Poland	Cohort study	Outpatient	Multidisciplinary rehabilitation program	97	✓	*Term:* described within baseline characteristics
Parker et al., 2023 [72]	United Kingdom	Cohort study	Home-based (telerehabilitation)	Structured pacing protocol	31	✓	*Term:* 'co-existing medical conditions'
Prabawa et al., 2022 [73]	Indonesia	Systematic review	NA	Physical rehabilitation therapy	NA	×	NA
Rodriguez-Blanco et al., 2023 [74]	Spain	RCT	Home-based (telerehabilitation)	Therapeutic exercise telerehabilitation protocol of physiotherapy	52	Excluded	*NA*
Romanet et al., 2023 [75]	France	RCT	Outpatient	Exercise training	60	✓	*Term:* 'comorbidities'
Rutsch et al., 2023 [76]	Germany	Mixed methods	Outpatient	Pneumological rehabilitation	221	✓	*Term:* 'comorbidities'
Sharma et al., 2022 [77]	India	RCT	Home-based (telerehabilitation)	Pulmonary tele-Rehabilitation	30	×	NA
Smith et al., 2023 [78]	United Kingdom	Cohort study	Home-based (telerehabilitation) and community site	Blended digital and community-based long-COVID-19 rehabilitation programme	601	✓	*Term:* 'comorbidities'
Stavrou et al., 2023 [79]	Greece	Feasibility randomised trial	Unclear	Exercise with virtual reality system	20	Excluded	NA

Szarvas et al., 2023 [80]	Hungary	Non-randomised trial	University department	Cardiopulmonary rehabilitation programme	68	✓	*Term:* 'pre-existing comorbidities'
Teixido et al., 2022 [81]	Germany	Cohort study (and narrative literature review)	Outpatient	Outpatient rehabilitative care	107	×	NA
Valverde-Martinez et al., 2023 [82]	Spain	Systematic review protocol	NA	Telerehabilitation	NA	×	NA
Volckaerts et al., 2023 [83]	Belgium	RCT protocol	Primary care	Pulmonary rehabilitation	NA	✓	*Term:* 'comorbidities'
World Physiotherapy, 2021 [84]	Multi-national authors	Consensus statement	NA	Safe rehabilitation approaches for people living with LC: physical activity and exercise	NA	×	NA

LC = Long Covid; LTC = long-term conditions; MLTC = multiple long-term conditions; NA = not applicable; NR = not reported; NYHA = New York Heart Association classification; RCT = randomised controlled trial

Eligibility criteria of studies that excluded individuals with pre-existing long-term conditions (*k=6*)

	Eligibility criteria	Justification / relevant discussion
Bouteleux et al., 2021 [40]	*Longitudinal observational study:*<br>"All patients referred in a participating centre with a medical prescription of ambulatory respiratory rehabilitation following suspected or confirmed SARS-CoV-2 infection were consecutively enrolled in this study" (pp.2)<br>"Standardised evaluation included collection of anthropometric data, work situation, **comorbidities** and clinical symptoms" (pp.2)<br>"**Patients had no comorbidities**" (pp.4)	Nil identified
Calvo-Paniagua et al., 2022 [43]*	"Exclusion criteria included: 1) patients with other post-COVID symptoms, e.g., gastrointestinal symptoms, anosmia, ageusia, or cognitive blurring; **2) evidence of pluri-pathology, i.e., more than two pre-existing medical comorbidities; 3, evidence of any medical co-morbidity, i.e., ischemic cardiopathy, cardiac or pulmonary insufficiency, potentially explaining fatigue or dyspnea; 4, presence of fatal medical co-morbidities e.g., cancer; 5, immunodeficient patients; 6, previous history of dementia or psychiatric disorders;** 7, patients with severe functional limitations (Barthel index score > 90); or 8) patients with cognitive problems." (pp.3)	Nil identified
Certain Curi et al., 2022 [44]	"Exclusion criteria comprise conditions in which fatigue is also a major complain such as **suspected or diagnosed chronic and/or neurological diseases (e.g., Parkinson's disease, amyotrophic lateral sclerosis, Alzheimer's disease); pre-existing, chronic diseases affecting the musculoskeletal system (e.g., fibromyalgia**)" (pp.24)	"Limitations of this protocol include threats to external validity and generalizability: **exclusion of participants with chronic neurologic and musculoskeletal conditions**" (pp.26)
Lobanov et al., 2022 [65]	"Exclusion criteria were as follows: 1. Patients with a febrile state or persistent subfebrile condition; 2. Severe unstable bronchial asthma or concomitant acute bronchial asthma; **3. Diagnosis of COPD – chronic obstructive pulmonary disease, exacerbation stage, established according to the current version of the GOLD guidelines, 2015 version; 4. Other chronic somatic, neurological or psychiatric conditions that may limit patient participation in the study**; 5. Participation in other clinical trials within one year prior to the inclusion in the study; 6. Poor patient cooperation; inability to understand informed consent" (pp.3)	"Additionally, rehabilitative protocol in the aquatic setting **could also properly address comorbidities** that can be present in post-COVID patients, such as obesity and other neurological and/or musculoskeletal problems" (pp.5)<br>"Innovative and efficient therapy paradigms that can address the COVID-19 infection outcomes, **paying particular attention to those connected to comorbidities**, should therefore be developed" (pp.5)
Rodriguez-Blanco et al., 2023 [74]	"The exclusion criteria were evaluated by a physician through a video call interview and were the following: participants who required hospital admission for COVID-19, cardiovascular or hypertension without medical treatment, **chronic lung or kidney diseases, chronic neurological or mental disorders, grade III osteoporosis**, and acute phase disorders (rheumatological and vertebral disc abnormalities). Patients who had suffered from **respiratory or musculoskeletal disease in the last 12 months (other than COVID-19)** and were not fully recovered, and those with signs of serious illness or red flags (night pain, severe muscle spasm, unintentional weight loss, imbalance of symptoms) without control by a health professional were also excluded" (pp.2)	Nil identified
Stavrou et al., 2023 [79]	"Inclusion criteria were at least 2 months post discharge, without fever for a 48-h period, stable, without supplemental O2, age ≥ 20 and ≤70 years, without absolute (unstable angina during the previous month and myocardial infarction during the previous month) and relative (resting heart rate >120 bpm, systolic blood pressure >180 mmHg, and diastolic blood pressure >100 mmHg) contraindications for the 6-min walk test (6MWT), body mass index <35 kg/m2, **comorbidity free (i.e., musculoskeletal disability, cardiorespiratory diseases, etc.),** color blindness, 6MWT ≥85% of predicted (16,17), and Montreal Cognitive Assessment questionnaire score, <26" (pp.2)	Nil identified

This study reported several single pre-existing LTC but excluded individuals with MLTC

Characteristics of reported physical rehabilitation interventions in applicable studies (*k=24*)

Study and year	Brief name of physical rehabilitation intervention	Rationale/ theory/ goal	Procedure	Timing post SARS-CoV-2 infection	Modes(s) and setting(s) of delivery	Frequency, intensity, and duration of PRI	Any tailoring to individuals' needs	Adjunctive intervention(s) / comparator(s)	Outcome domains reported
Altmann et al., 2023 [36]	Rehabilitation program - endurance and resistance muscular training	NR	Endurance training as possible with fatigue symptoms (bicycle ergometer exercise training, aqua training and terrain training with self-management using pretested or calculated heart rate or perceived exertion using the Borg Scale).	Admitted mean 10.1 months (4-20) after COVID-19 infection.	Supervised: face-to-face<br><br>*Setting:* inpatient	*Frequency:* NR<br>*Intensity:* NR<br>*Duration*: 25 minutes per session, 4-5 weeks	Management of comorbidity as needed. Psychological counselling and psychiatric appointment as needed. Special attention was given to social needs after discharge at home or at work.	*Adjunctive interventions:* multimodal respiratory therapy, psychological assistance, and educational measure	Exercise capacity<br>HRQoL<br>Disability<br>Other: minimal peripheral oxygen saturation on exertion, inspiratory muscle strength, fit for work.
Barbara et al., 2022 [37]	Exercise rehabilitation	To increase cardiorespiratory and musculoskeletal fitness.	Aerobic exercise was followed by nine major muscle group resistance exercises (for the lower extremity: leg extension/flexion, abduction/adduction, and leg press; for the upper extremity: push-up/pull-down; for the core muscles; abdomen, back).	NR	Supervised: face-to-face<br><br>*Setting:* outpatient	*Frequency:* x3/week.<br>*Intensity:* aerobic - 80% lactate threshold according to VO2 peak test results, resistance - 40% 1RM.<br>*Duration:* aerobic - 30 mins increasing to 60 mins, resistance - 90 mins, 8 weeks.	Tailored according to intensity.	NA	Exercise capacity<br>Functional ability
Besnier et al., 2022 [39]	Cardiopulmonary rehabilitation	NR	Aerobic exercise will be performed on a cyclo-ergometer. Strengthening exercises will be performed with weight machines, free weights and/or elastic bands, and will target large muscle groups.	NA (protocol)	Supervised: face-to-face<br><br>*Setting*: outpatient	*Frequency:* x3/week<br>*Intensity:* aerobic - individualised according to first ventilatory threshold, strength - *starting at an intensity of 40% of the maximum strength*<br>*Duration:* aerobic - 30 mins, *strength – 3 sets of 10 reps,* 8 weeks.	Individualised according to the F.I.T.T. principles (frequency, intensity, time, and type).	*Adjunctive interventions*: breathing exercises – pursed lip breathing and inspiratory muscle training<br><br>*Comparator*: control group – no intervention	Exercise capacity<br>Functional ability<br>HRQoL<br>Disability<br>Other: respiratory capacity, stress, sleep quality, Long Covid symptoms, coagulation, inflammatory and antioxidant profile and brain health

Brough et al., 2022 [42]	Physiotherapy and mind-body interventions	To address strength, balance, endurance, and co-ordination and help restore muscle tone and joint support.	Simple functional activity such as sit to stand and step work for stair practice. It also included some balance work and coordination for core stability. Some light resistance work for the upper and lower limb for individuals that had returned to a higher level of activity prior to attending.<br><br>QiGong and seated yoga included as part of programme of mind-body interventions.	NR	Supervised (individual): face-to-face<br><br>*Setting:* community site	*Frequency:* NR<br>*Intensity:* NR<br>*Duration:* up to 45 min sessions, 4- or 6-week programme	Each session was adapted to the individual need.	*Adjunctive interventions:*<br>- Psychoeducation and mind-body interventions<br>- Craniosacral therapy<br>- Aftercare	HRQoL<br>Disability<br>Other: blood pressure and oxygen saturation
Calvo-Paniagua et al., 2022 [43]	Telerehabilitation progam - physical activity	NR	Specific physical conditioning targeting the spine, respiratory, core and lower and upper extremity muscles performing aerobic exercise trainings, active mobilizations and motor control exercises.	The mean duration of the post-COVID symptoms was 4.7 ±0.5 (SD) months after hospital discharge.	Supervised: remote (video-conferencing)<br><br>*Setting:* Primary care	18 sessions x 40 mins over 7 weeks (3 per week)	NR	*Adjunctive interventions*:<br>Patient education<br>Airway clearing<br>Breathing exercise interventions	Exercise capacity<br>HRQoL<br>Disability<br>Other: changes in oxygen saturation and heart rate during 6MWT
Compagno et al., 2022 [46]	Multidisciplinary rehabilitation program - physical training	Physical reconditioning, reducing symptoms, improving physical fitness and psychological parameters.	Endurance training was carried out with the use of cardio machines, such as cycle ergometer and treadmill. Aerobic exercise was followed by resistance strength training, conducted with the use of compressed-air isotonic machines (pectoral machine, lower-back, leg press, leg extension, adductor machine, deltoids press). At the end of the training session, stretching activities were performed.	After a mean of 3 months (range 1-6 months) from the resolution of acute COVID-19 infection.	Supervised: face-to-face<br><br>*Setting:* outpatient	*Frequency*: x3/week.<br>*Intensity*: aerobic - corresponding to 60-80% of VO2 peak, resistance – 30-50% of the 1-RM, registered during CPET.<br>*Duration*: x90 min sessions.	Prescription of intensity.	*Adjunctive intervention*: psychosocial treatment	Exercise capacity<br>Functional ability<br>HRQoL<br>Other: body composition, safety and feasibility of the program, anxiety and depression
Daynes et al., 2023 [47]	COVID rehabilitation - face-to-face exercise rehabilitation	To improve exercise capacity and symptoms.	Symptom-titrated exercise therapy:<br>- aerobic exercise (treadmill/ground walking, cycling on a cycle ergometer) and resistance exercise training (upper and lower limb strength exercises),<br>- patients will be asked to perform home-based exercise sessions which mimic the supervised sessions: three aerobic exercise sessions and one resistance exercise session	NA (protocol)	Supervised: face-to-face, and unsupervised (home-based) components.<br><br>*Setting:* outpatient and home-based	*Frequency*: up to x5/week (x2 supervised).<br>*Intensity*: aerobic - 80% of ISWT speed.<br>*Duration*: 90-120 mins per session, 8 weeks.	Individualised symptom titrated programme; use of predicted VO_2 max determined by ISWT. Exercise intensity and duration tailored to individuals' current abilities.	*Adjunctive interventions*: education and self-management<br><br>*Comparator:* Digital rehabilitation (unsupervised self-guided app with telephone reviews).	Exercise capacity<br>Functional ability<br>HRQoL<br>Physical activity<br>Frailty<br>Disability<br>Other: fatigue (FACIT-FS), anxiety and depression, breathlessness (D12), cognitive impairment

			per week which are recorded in a self-reported diary.						(MoCA), hyperventilation (Nijmegen), blood markers, muscle biopsies.
deOliveira et al., 2023 [49]	Multicomponent exercises	To improve functional mobility.	-Walking<br>-Strength exercises (7 exercises with 2 sets of 10 repetitions for upper and lower limb muscle groups, using elastic bands, free weights and ankle weights, with weight progressively increased according to the participant's ability);<br>-Balance: postures with a gradual decrease in the support base, dynamic movements that disturb the post-stress center of gravity of muscle groups, dynamic movements when performing secondary tasks individually;<br>-Relaxation	NR	Supervised (group): face-to-face<br><br>*Setting:* unclear	*Frequency*: twice weekly<br>*Intensity:* NR<br>*Duration*: 60 mins per session, 12 weeks	NR	*Comparator:* Control group - no training	Exercise capacity<br>Functional ability<br>HRQoL
Estebanez-Pérez et al., 2022 [51]	Digital physiotherapy intervention	To improve functional capacity.	Digital physiotherapy interventions could include personalised recommendations for each patient like walking, jogging or swimming added to the supervised digital interventions based on individual patient needs, starting at a low intensity and duration and increasing gradually. Progressive strength training was recommended.	NR	Supervised: remote (video-conferencing), and unsupervised components.<br><br>*Setting:* home-based (telerehabilitation)	*Frequency*: 1:1 synchronous limited to 1 session per day (number of synchronous sessions determined by initial evaluation), 3-5 digital intervention sessions/week recommended, 3-5 sessions/week of progressive strength training recommended.<br>*Intensity*: progressive strength - 2-min intervals increasing load by 5-10% per week.<br>*Duration*:45-40 mins max per session (1:1 synchronous), 20-30 min duration	Individualised and customise exercise programme.	NA	Exercise capacity<br>Functional ability

						recommended (digital intervention sessions), 4 weeks, minimum 4 weeks for progressive strength training recommended.			
Fowler-Davis et al., 2021 [53]	Virtual multidisciplinary rehabilitation intervention	NR	Exercise prescription comprised short walks and the repeated sit to stand exercise.<br>Qualitative case description: "*The intervention included a ten-minute health walk with support for breathlessness, joint pain and fatigue*" (pp.9).	NR	Unsupervised<br><br>*Setting:* home-based (telerehabilitatio n)	*Frequency:* NR<br>*Intensity:* NR<br>*Duration*: NR	Intervention of each participant was tailored to their current physical activity levels, medical history and current problems.	*Adjunctive interventions:* virtual clinic activity sessions.	Functional ability<br>HRQoL<br>Disability<br>Other: fatigue.
Frisk et al., 2023 [54]	Micro-choice based rehabilitation - physical activity / exercise training	*From protocol (Kvale et al., 2021):*<br>to increase exercise capacity and the restoration of trust in one's own body.	*From protocol (Kvale et al., 2021):*<br>Physical activity sessions - instructions to attempt making the physical activity relevant for their own challenges and fit into their projects of 'breaking patterns of symptom regulation'. For post–COVID-19 patients, the physical training will be a mix of high- and low-intensity training.	Time to rehabilitation after confirmed COVID-19 (months): mean 10.2 ±4.8 (SD).	Supervised (group): face-to-face<br><br>*Setting:* outpatient	*Frequency:* NR<br>*Intensity:* mix of high and low.<br>*Duration*: 3 consecutive days (8.30-16.00) (with 7 day and 3 month follow up).	Individually tailored.	*Adjunctive interventions:* education exercise on breaking inflexible patterns, mindfulness, feedback and coaching, food and meal habits, pharmacist. medication list review	Exercise capacity<br>Functional ability<br>HRQoL<br>Disability<br>Other: fatigue, sick leave, dyspnoea, lung function, ongoing psychiatric symptoms, work and social adjustment scale.
Grishechkina et al., 2023 [55]	Multidisciplinary rehabilitative interventions: balance and motor training, and a tailored aquatic exercise intervention	NR	Program of aquatic exercises conducted in a bromine sodium chloride water.	The mean time since the onset of the acute COVID-19 infection was 6.5±2.5 months.	Supervised: face-to-face<br><br>*Setting:* spa	*Frequency:* NR<br>*Intensity:* NR<br>*Duration*: 7-8 sessions.	Tailored protocol of aquatic exercises.	*Adjunctive interventions*: respiratory exercises, social integration training, neuropsychologica l sessions, LASERtherapy, magnetotherapy.<br><br>*Comparator 1:* climatotherapy, traditional oriental medicine methods, exercise therapy, LASERtherapy and magnetotherapy.	Disability<br>Mortality<br>Hospitalisation<br>Other: number of ambulance calls due to exacerbation of LC symptoms, need for specialist consultations.

								Comparator 2: respiratory and motor exercise therapy, physiotherapy combined with inhalation of mineral water, balneotherapy with dry carbon dioxide baths and magnetotherapy.<br><br>*Comparator 3:* self-training and home-based physical exercise.	
Hentschel et al., 2022 [58]	Outpatient rehabilitation services	NR	NR<br>*(Codes for physical, occupational, respiratory, and cognitive therapies, combined with an indicator for outpatient setting for provision of services, were used to identify utilization and type of post COVID-19 rehabilitation therapies).*	NR	*Mode:* NR<br><br>*Setting:* outpatient	NR	NR	*Comparator:* matched controls (confirmed positive covid case but did not access rehab).	Other: receipt of outpatient rehabilitation services within 6 months of COVID-19 diagnosis and incidence of post COVID-19 condition.
Jimeno-Almazán et al., 2022 [59]	Multicomponent exercise program adapted from the ACSM guidelines for chronic obstructive pulmonary disease and cardiovascular disease	To improve physical and mental status compared to the conventional self-management recommendations.	Supervised resistance training combined with aerobic training (moderate intensity variable training), plus a third day of monitored light intensity continuous training.	NR	Supervised: face-to-face<br><br>*Setting:* outpatient	*Frequency:* x3/week (2 days resistance, 1-day light continuous training).<br>*Intensity:* resistance - 50% 1PM 3 sets 8 reps 4 exercises, aerobic - moderate intensity variable training 4-6 x 3-5min at 70-90% heart rate reserve (HRR)/ 2-3min at 55-65% HRR, 3rd day light intensity continuous training - 65-70% HRR.<br>*Duration:* 8 weeks, 3rd day light intensity session	Tailored according to intensity.	*Comparator:* control group (followed the WHO guidelines for rehabilitation after COVID-19).	Exercise capacity<br>Functional ability<br>HRQoL<br>Physical activity<br>Disability<br>Other: anxiety and depression, fatigue, ME/CFS symptoms, lung function, body composition.

						30-60mins.			
Jimeno-Almazán et al., 2023 [60]	Multicomponent exercise program based on concurrent training	In addition to regaining physical function, exercise could relieve symptom burden, improve health-related quality of life and, through its anti-inflammatory properties and its enhancing effect on the immune system, improve patients after COVID-19 infection.	Resistance and endurance supervised sessions (resistance training comprised squat, bench press, deadlift, bench pull).	Reported as weeks of symptoms: mean 39.3 ± 23.3 (SD).	Supervised, face-to-face<br><br>*Setting:* outpatient	*Frequency*: x3 sessions per week (2 days resistance training).<br>*Intensity*: resistance - 50% 1PM 3 sets 8 reps 4 exercises, moderate intensity variable training - 4-6 x 3-5min at 70-80%heart rate reserve (HRR)/ 2-3 min at 55-65% HRR, one day light intensity.<br>*Duration*: 8 weeks.	Individualised intensity: HR max and VAS used to tailor exertion.	*Comparator 1:* inspiratory muscle training.<br><br>*Comparator 2:* multicomponent exercise program and inspiratory muscle training.<br><br>*Comparator 3:* control group (following the WHO guidelines for post-COVID-19-related illness rehabilitation).	Exercise capacity<br>Functional ability<br>HRQoL<br>Disability<br>Other: fatigue, health status, anxiety and depression, number of symptoms.
Kupferschmitt et al., 2022a [62]	Specialised rehabilitation: pneumological, cardiological, psychosomatic, neurological	To favourably influence LC symptoms.	Varied across groups/sites: exercise therapy, monitored ergometer training, strength training, vibration platform training, back school, balance training, stair climbing, spinal gymnastics, nordic walking, aqua fitness, walking, stamina training, coordination training.	NA (protocol)	Supervised (face-to-face)<br><br>*Setting:* outpatient	Varied across groups/sites: -<br>*Frequency*: x1-5/week.<br>*Intensity:* NR<br>*Duration*: 30-90 mins.	NR	*Adjunctive interventions:* psychoeducation, relaxation, psychotherapy, respiratory therapy, cognitive training, creative therapy.<br><br>*Comparator:* control group (no intervention).	Exercise capacity<br>Functional ability<br>Disability<br>Other: COVID-19 symptoms, work ability index, anxiety/depression, life skills, fatigue, avoidance-endurance, cognitive assessment, attention, post-exertional malaise, pulmonary function tests/spiroergometry/blood gas/echo.
Nopp et al., 2022 [70]	Multi-professional and individualized rehabilitation	NR	Individualised endurance and strength training.	Time to rehabilitation after confirmed COVID-19 mean 4.4 ±2.0 (SD) months.	*Mode:* unclear<br><br>*Setting:* outpatient	*Frequency*: x3/week.<br>*Intensity:* NR<br>*Duration*: 3-4 hour sessions, 6 weeks.	Individualised rehabilitation (no further detail provided).	*Adjunctive interventions*: inspiratory muscle training, education, psychosocial counselling, nutritional	Exercise capacity<br>Functional ability ; HRQoL<br>Disability<br>Other: Borg dyspnoea, fatigue,

								education, smoking cessation sessions.	pulmonary function.
Ostrowska et al., 2023 [71]	Multidisciplinary rehabilitation program - physical training	To relieve dyspnoea, psychological distress, and improve participation in rehabilitation, physical function, and quality of life.	Physical training (aerobic, resistance, and breathing exercises).	NR	Supervised: face-to-face<br><br>*Setting:* outpatient	*Frequency*: x3/week.<br>*Intensity:* NR<br>*Duration*: 90 min session, 6 weeks.	Comprehensive medical and physiotherapeutic assessment was performed to individualise the multidisciplinary rehabilitation program.	*Adjunctive intervention:* education, group psychotherapy.	Exercise capacity<br>Functional ability<br>Disability<br>Other: fatigue, body composition.
Parker et al., 2023 [72]	WHO Borg CR-10 pacing protocol	To reduce PESE episodes.	Implementation of the WHO Borg CR-10 5-phase pacing protocol. The phases begin with a "preparation for return to activity" phase, RPE 0−1, where breathing exercises and gentle stretches are suggested activities, and concludes with "return to baseline exercises," RPE 8−10, where patients should be able to complete pre-COVID-19 activities. The clinician administered PESE and C19-YRS questionnaires weekly and advised target activity level.	The median self-reported duration of PCS was 17 months (IQR: 12−27 months).	Unsupervised<br><br>*Setting:* home-based (telerehabilitation)	*Frequency*: weekly telephone calls.<br>*Intensity*: phase 1 RPE 0-1, phase 2 RPE 2-3, phase 3 RPE 4-5, phase 4 RPE 5-7, phase 5 RPE 8-10.<br>*Duration*: 6 weeks.	Tailoring based on weekly PESE and C19-YRS questionnaires.	NA	HRQoL<br>Other: PESE episodes.
Romanet et al., 2023 [75]	Exercise training rehabilitation	NR	Continuous endurance training: endurance training and exercise tolerance were evaluated using a cycle ergometer.<br>Muscle strength training: strengthening of the lower limbs was prioritised, but additional exercises for the upper limbs and core were also included. Exercises included leg presses, leg curls, and leg extensions using devices such as steppers, rowing machines, treadmills, and weights. Individual exercise choices were at the discretion of the physiotherapist, but muscle fatigue had to be felt at the end of each set.	NR	Supervised: face-to-face<br><br>*Setting:* outpatient	*Frequency*: x2/week. *Intensity*: started at 60-70% of max peak power and Borg 4-6 (continuous endurance training), power intensity was adjusted according to each participant's progress until the target heart rate and dyspnoea were reached.<br>*Duration*: 60 min sessions, muscle strengthening training 4 sets of 6-12 reps, 10 weeks / 90 days.	Use of 6MWT to determine target HR.	*Comparator*: standard physiotherapy.	HRQoL<br>Disability<br>Other: multidimensional dyspnoea.
Rutsch et al.,	Physical	*From protocol*	*From protocol (Rutsch 2021):*	Unclear	Supervised:	*Frequency:* NR	NR	*Adjunctive*	Functional ability

2023 [76]	rehabilitation	*(Rutsch 2021):* Intended to improve respiratory distress, respiratory muscle strength, pulmonary resilience, psychological impairments and/or stress-induced mental disorders of the disease.	5 different clinics with differing descriptions:<br>- General strengthening, mobilisation and normalisation of respiratory functions.<br>- Therapy contents include physical and balneotherapy, physiotherapy and exercise therapy, occupational therapy and psychological support.<br>- A multi-modal and holistic treatment approach during pneumological rehabilitation.	(*On average, the rehabilitators started the healing procedure five months after their infection with SARS-CoV-2*)	face-to-face<br><br>*Setting:* outpatient	*Intensity:* NR<br>*Duration*: 3-5 weeks.		*intervention*: respiratory gymnastics with respiratory muscle training, group discussions (as required), relaxation training and supportive psychological counselling to cope with trauma, fear or depression.<br><br>*Comparator:* control group (COPD and asthma bronchial patients) - treatments comparable to that received by those after COVID-19 (physical rehabilitation).	HRQoL<br>Disability<br>Other – COVID-related symptoms and associated life events, fatigue, depression/anxiety/stress, COPD assessment test, work ability, subjective prognosis of employment, performance in different areas of life (areas of work, everyday life and leisure), sociodemographic data.
Smith et al., 2023 [78]	LC rehabilitation programme	Aims to improve symptoms of LC, functional capacity, personal well-being and HRQoL.	Access to a web-based rehabilitation hub with the on-demand exercise sessions, webinars, and resources - physical copy of a rehabilitation journal with information, advice, and activities to support recovery.<br>Exercise sessions including a group session, a pre-recorded session and self-directed session.<br>Activity sessions included a combination of cardiovascular, strength-based and mobility exercises.	Mean period of 9.8 ±5.0 (SD) months between COVID-19 diagnosis and commencing the rehabilitation programme.	Supervised (group): first 6 weeks remote, second 6 weeks incorporated face-to-face sessions.<br><br>*Setting:* home-based (telerehabilitation)	*Frequency*: x3/week, weekly telephone call<br>*Intensity*: prescribed according to exercise capacity testing.<br>Duration: 45 min sessions, 12 weeks.	Target exercise intensity and volume, as well as movement complexity, range of motion, and stability were prescribed according to the participants functional capacity and physical fitness which was recorded at baseline (using the Duke Activity Status Index and Sit-to-Stand test).	NA	Functional ability ; Health-related quality of life;<br>Other: dyspnoea, well-being, illness burden (GP consultations, outpatient visits, sick days).
Szarvas et al., 2023 [80]	Intensive pulmonary rehabilitation	To assist recovery and regaining physical and mental (emotional, psychological, and social well-being) health.	Group exercise intervention: controlled breathing, chest mobility-enhancing and muscle strengthening exercises with own body weight and dumbbells.<br>Low intensity individual training: gym bike, treadmill or arm ergonometer.	Unclear<br>(*The rehabilitation programme started usually at least 3.5 months after the COVID-19 infection*)	Supervised (group): face-to-face<br><br>*Setting:* university department	*Frequency*: 2/3 times daily.<br>*Intensity*: group intervention HR, individual training 'low intensity'.<br>*Duration*: 30 mins/session, 14 days.	Age, comorbidities, and current conditions were taken into account.	*Adjunctive intervention:* respiratory muscle training.	Exercise capacity<br>Functional ability ; HRQoL<br>Disability<br>Other: respiratory function, chest kinematics,

									breath-holding test.
Volckaerts et al., 2023 [83]	Pulmonary rehabilitation	To improve exercise capacity, symptoms, physical activity and sleep.	Muscle strength training and endurance training: phased exercise programme (1-5) based on RPE scores.<br>1 (RPE 6-8): prepare to resume physical activity using breathing exercises, stretching, balance exercises, walking.<br>2 (RPE 6-11): low intensity exercises (walking, light yoga and light ADL tasks) with gradual increase of 10–15 min extra per day.<br>3 (RPE 12-14): medium-intensity aerobic exercise and more challenging strength exercises (e.g., interval exercise).<br>4 (RPE 12-14): more complex aerobic exercises of medium intensity and more challenging with strength exercises that also include coordination and functional elements.<br>5 (RPE>15): Return to normal 'baseline' exercise level.	NA (protocol)	Supervised: face-to-face<br><br>*Setting:* primary care	*Frequency*: x3/week.<br>*Intensity*: based on RPE scores.<br>*Duration*: 12 weeks.	Tailored according to intensity.<br>Treatment components must be adapted to the patients need.	*Adjunctive interventions:* information and education, respiratory muscle training and breathing exercises.<br><br>*Comparator*: control group (no intervention).	Exercise capacity<br>Functional ability ; HRQoL<br>Physical activity<br>Disability<br>Other: pulmonary function tests, CAT, dyspnoea, Nijmegen, anxiety and depression, sleep efficiency, work productivity.

C19-YRS = COVID-19 Yorkshire rehabilitation scale; ISWT = incremental shuttle walk test; IQR = interquartile range; LC = Long Covid; NR = not reported; HRQoL = health-related quality of life; ME/CFS = myalgic encephalomyelitis/chronic fatigue syndrome; RPE = rating of perceived exertion; PCS = post-COVID-19 syndrome; PESE = post exertional symptom exacerbation; PRI = physical rehabilitation intervention; SD = standard deviation; VO_2 max = peak oxygen uptake; 6MWT = 6-minute walk test; WHO = World Health Organisation; highlighted studies are those that reported number of pre-existing LTC enabling identification of those with MLTC

Outcome measure domain reporting (*k=24*)

	1	2	3	4	5	6	7	8	9	10	11	12	13	14	15	16	17	18	19	20	21	22	23	24
Exercise capacity	✓	✓	✓		✓	✓	✓	✓	✓		✓			✓	✓	✓	✓	✓				✓	✓	✓
Functional ability		✓	✓			✓	✓	✓	✓	✓	✓			✓	✓	✓	✓	✓			✓	✓	✓	✓
HRQoL	✓		✓	✓	✓	✓	✓	✓		✓	✓			✓	✓		✓		✓	✓	✓	✓	✓	✓
Physical activity							✓							✓										✓
Frailty							✓																	
Disability	✓		✓	✓	✓		✓			✓	✓	✓		✓	✓	✓	✓	✓		✓	✓		✓	✓
Mortality												✓												
Hospitalisation												✓												

1 Altmann 2023 [36]
2 Barbara 2022 [37]
3 Besnier 2022 [39]
4 Brough 2022 [42]
5 Calvo-Paniagua 2022 [43]
6 Compagno 2022 [46]
7 Daynes 2023 [47]
8 deOliveira 2023 [49]
9 Estebanez-Pérez 2022 [51]
10 Fowler-Davis 2021 [53]
11 Frisk 2023 [54]
12 Grishechkina 2023 [55]
13 Hentschel 2022 [58]
14 Jimeno-Almazán 2022 [59]
15 Jimeno-Almazán 2023 [60]
16 Kupferschmitt 2022 [62]
17 Nopp 2022 [70]
18 Ostrowska 2023 [71]
19 Parker 2023 [72]
20 Romanet 2023 [75]
21 Rutsch 2023 [76]
22 Smith 2023 [78]
23 Szarvas 2023 [80]
24 Volckaerts 2023 [83]

Time post SARS-CoV-2 infection at point of intervention delivery (*k=9*)

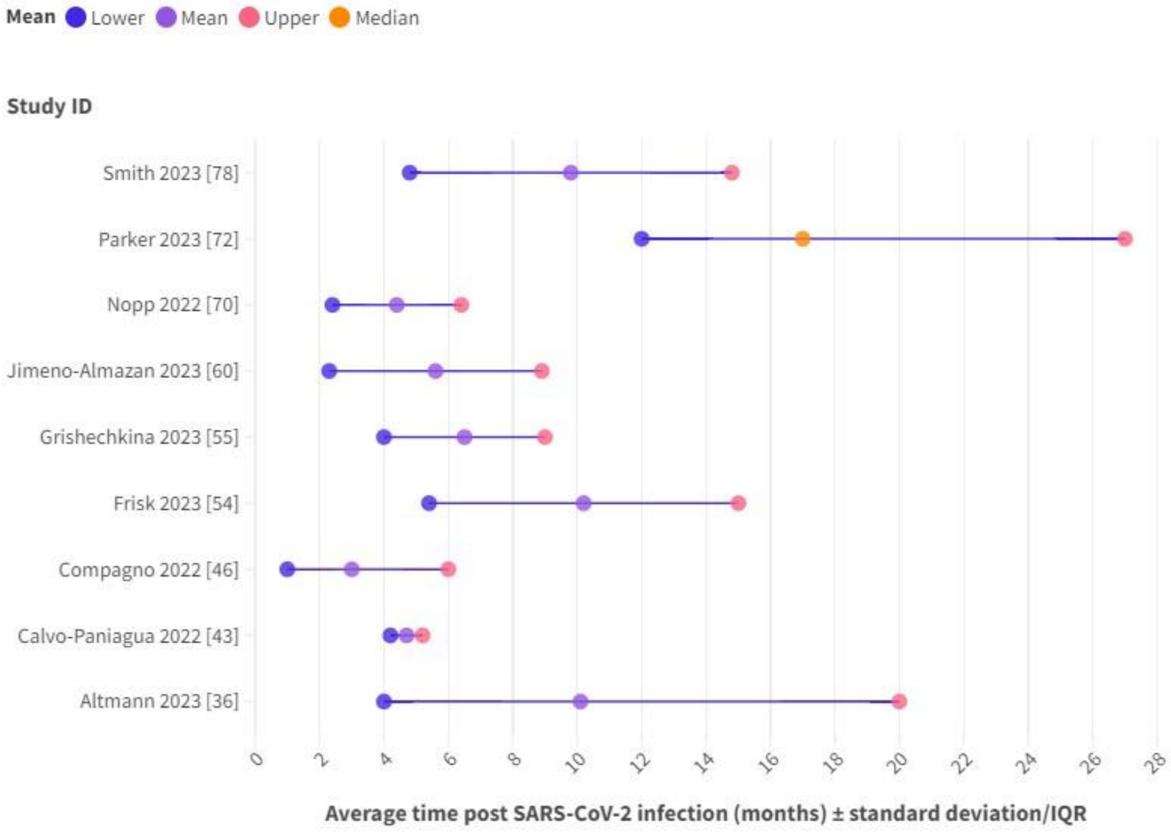

Supplement: Supplementary file 1 [file ERR-0123-2024.SUPPLEMENT.pdf]
